# Cross-modal integration of emotions in the chemical senses

**DOI:** 10.3389/fnhum.2013.00883

**Published:** 2013-12-20

**Authors:** Moustafa Bensafi, Emilia Iannilli, Valentin A. Schriever, Johan Poncelet, Han-Seok Seo, Johannes Gerber, Catherine Rouby, Thomas Hummel

**Affiliations:** ^1^CNRS UMR5292, INSERM U1028, Lyon Neuroscience Research Center, University LyonLyon, France; ^2^Smell and Taste Clinic, Department of Otorhinolaryngology, University of Dresden Medical SchoolDresden, Germany; ^3^Department of Food Science, University of ArkansasFayetteville, AR, USA; ^4^Department of Neuroradiology, University of Dresden Medical SchoolDresden, Germany

**Keywords:** olfaction, trigeminal, emotion, fMRI, congruency

## Abstract

Although the brain structures involved in integrating odorant and trigeminal stimuli are well-documented, there is still a need to clarify (1) how emotional response is represented in the human brain during cross-modal interaction between odors and trigeminal stimuli, and (2) whether the degree of congruency between the two types of stimuli influences these emotional responses and their neural processing. These questions were explored combining psychophysics, event-related potentials (ERP) and fMRI in the same group of 17 subjects under a “congruent condition” (intranasal carbon dioxide mixed with the smell of orange, a combination found in soda drinks, for example), and an “incongruent condition” (intranasal carbon dioxide mixed with the smell of rose, a combination not encountered in everyday life). Responses to the 3 constituent stimuli (carbon dioxide, orange, and rose) were also measured. Hedonic and intensity ratings were collected for all stimulations. The congruent bimodal stimulus was rated as more pleasant than the incongruent. This behavioral effect was associated with enhanced neural activity in the hippocampus and anterior cingulate gyrus, indicating that these brain areas mediate reactivation of pleasant and congruent olfactory-trigeminal associations.

## Introduction

Chemosensation comprises three main sensory modalities: olfaction and gustation, involved in discrimination and identification of, respectively, odorant and tastant stimuli, and the trigeminal system involved in detecting the irritating, fresh or painful component of chemosensory stimuli [see Lundstrom et al. (2011) for a review]. Past and current studies have detailed the functioning of each of these systems (Anderson et al., 2003; Small et al., 2003; Boyle et al., 2007b; Hummel et al., 2009a,b), but their interactions (although numerous and very close) have been much less studied (Small and Prescott, 2005; Boyle et al., 2007a; Bensafi et al., 2012). Moreover, one important transversal aspect is the strong emotional component of chemosensory perception. Firstly, a particular odor, taste or trigeminal stimulus can provide an early warning of toxic substances (spoiled or toxic food, industrial pollutants). Secondly, olfaction, taste and the trigeminal system combine to play a major role in hedonic experience: orangeade, with its orange odor, sweet taste and fresh gaseous components, can be best appreciated on a hot summer's day or after a sports effort.

The chemical senses thus provide a special window onto the cross-modal integration of emotion: chemosensory stimuli are mixtures of various compounds stimulating the olfactory, gustatory and trigeminal systems; each system may evoke particular affective states. The mechanisms and brain structures involved in the neural integration of odors and tastes have been well-documented in the last decade (Dalton et al., 2000; De Araujo et al., 2003; Small et al., 2004; Small and Prescott, 2005), but there is still a need to understand how emotional responses are represented in the human brain during cross-modal interaction of odors and trigeminal stimuli. Psychophysical and neuroimaging studies have highlighted the role of congruency in this cross-modal integration. Regarding food in particular, congruency has been defined as the extent to which sensory stimuli can appropriately combine in eating or drinking a given foodstuff (Schifferstein and Verlegh, 1996). Past and recent studies suggest that congruency is a key factor in modulating the cross-modal integration of chemosensory stimuli, especially when the sensory cues belong to the same object.

Schifferstein and Verlegh (1996), studying odor-taste interactions, showed that the pleasantness of odor-taste mixtures correlates positively with the degree of congruency between the two types of stimulus. For these authors, two components need to form a harmonious (or congruent) combination in order to be pleasant.

Pleasantness, congruency and harmony are nevertheless linked to familiarity, which may explain why adding an unpleasant stimulus (salt to chocolate, or CO_2_ to a beverage) can increase the overall pleasantness of the combination. Thus, a less rewarding stimulus (such as salt or pepper in chocolate) or an intrinsically painful stimulus (such as CO_2_ in a beverage) becomes part of the integrated percept of a familiar food. As suggested by Rozin and colleagues, the memory of a food may inhibit the painful or warning value of the trigeminal input, and even make it desirable (Rozin et al., 1982).

On the neural level, De Araujo and colleagues showed that a congruent odor-taste combination (strawberry/sucrose) was perceived as more pleasant than an incongruent one (strawberry/monosodium glutamate) and that increasing congruency correlated positively with antero-medial orbitofrontal activity (De Araujo et al., 2003). Likewise, Small et al. observed that a congruent odor-taste mixture (vanilla/sweet) was perceived as more pleasant than an incongruent mixture (vanilla/salt) (Small et al., 2004). Moreover, the congruent odor-taste mixture induced greater activation than its components in the anterior cingulate cortex, insula, posterior orbitofrontal cortex, prefrontal cortex and parietal cortex, whereas the same brain areas were not activated in a similar comparative analysis of perception of the incongruent odor-taste mixture.

Such a congruency effect was also reported in odor-vision interaction. Gottfried and Dolan showed that congruent pairs of visual and olfactory stimuli were detected faster than incongruent pairs, and activated the rostro-medial orbitofrontal cortex and the hippocampus (Gottfried and Dolan, 2003). Even color has an effect on the perception of smells. In an fMRI study, Osterbauer and colleagues scanned human subjects exposed to smells and colors, in isolation and in congruent or incongruent combinations: activity in the posterior orbitofrontal cortex and insula increased as a function of the congruency of the smell-color pairs (Osterbauer et al., 2005). Using olfactory event-related potentials, Seo et al. showed that a congruent abstract visual symbol enhanced the intensity of the smell of rose compared to presentation of no symbol; it increased the pleasantness of rose odor and the unpleasantness of an unpleasant odor; and congruent symbols induced significantly higher amplitudes and shorter latencies in the N1 component of olfactory event-related potentials than did incongruent symbols (Seo et al., 2010).

Finally, Seo and Hummel extended this effect of congruency to odor-sound integration, demonstrating that even auditory cues can modulate odor pleasantness. Subjects were presented with congruent, incongruent or neutral sounds before and during the presentation of a smell: the olfactory stimuli were rated more pleasant in the presence of a congruent than an incongruent sound (Seo and Hummel, 2011).

Thus, congruency affects perception at different levels of processing, from detection (Gottfried and Dolan, 2003) to intensity (Seo et al., 2010) or pleasantness (Schifferstein and Verlegh, 1996; De Araujo et al., 2003; Small et al., 2004; Seo and Hummel, 2011). In addition, this perceptual modulation is associated with neural changes in a set of sensory and heteromodal areas, including the orbito-frontal cortex, cingulate cortex, insula, hippocampus, prefrontal cortex, and parietal cortex.

The first aim of the present study was to examine the influence of congruency on the (1) pleasantness and intensity of olfacto-trigeminal mixtures and (2) brain activity in the above-cited central structures in response to bimodal odor-trigeminal stimulation.

Moreover, congruency seems to affect the temporal processing of chemosensory cross-modal integration, as suggested by the chemosensory event-related potential (CSERP) study by Seo et al. (2010). In human adults, CSERPs usually include two main components: (1) a negative component (N1) at around 400 ms; and (2) a late positive component (P2) at around 600 ms. Congruency has been shown to affect N1 latency and amplitude; its effect on the P2 component, however, is not clear. P2 amplitude increases as a function of emotional intensity and P2 latency has been shown to decrease with odor pleasantness (Pause and Krauel, 2000; Lundstrom et al., 2006; Poncelet et al., 2010).

The second aim of the present study was to test the influence of congruency on both the N1 and P2 CSERP components in response to odor-trigeminal stimuli. Psychophysics, fMRI and electroencephalography were combined in the same subjects under congruent and incongruent conditions particularly relevant to food: in the “congruent” condition, intranasal carbon dioxide was mixed with the smell of orange (a combination found in soda drinks), and in the “incongruent condition” intranasal carbon dioxide was mixed with the smell of rose (a combination not encountered in everyday life). Responses to the 3 unimodal stimuli (carbon dioxide, orange and rose) were also measured. Pleasantness and intensity ratings and hemodynamic responses (fMRI) from all five conditions were measured. After functional imaging, EEG responses (CSERP) to these conditions were collected from all participants.

## Materials and methods

### Subjects

Participants were 17 right-handed volunteers; mean age: 23.58 ± 1.97 years; 4 male, 13 female. They received 20 Euros for participation. The recording procedure was explained in great detail to the subjects, who provided written consent prior to participation. The study was conducted according to the Declaration of Helsinki and was approved by the ethics committee of the University of Dresden. Instructions consisted of an explanation of the experimental design, which included functional, anatomical and EEG sessions. Subjects were instructed to not move during the fMRI experiment. Detailed medical history combined with nasal endoscopy of the nasal cavity and odor perception assessment by the “Sniffin' Sticks” test (Hummel et al., 1997) ascertained that subjects were in good health and had normal sense of smell.

### Stimuli and olfactometer

**Figure 1:**
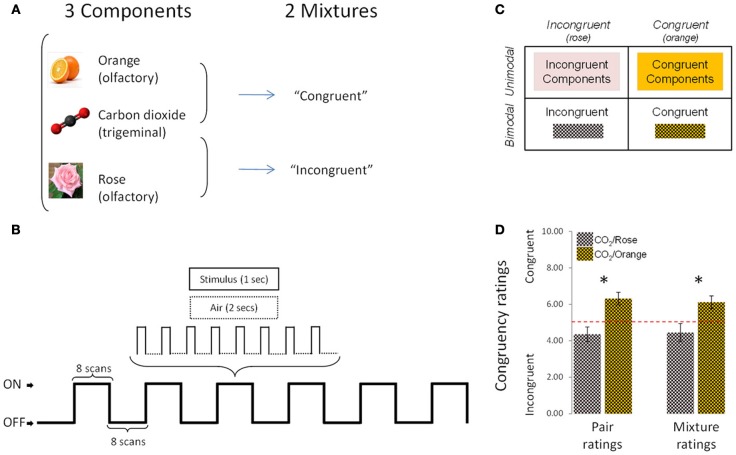
**Experimental design, protocol and congruency ratings**. **(A)** The experimental design comprised 5 conditions (3 individual components and 2 mixtures). **(B)** Schematic representation of the experimental protocol used during the fMRI sessions. **(C)** 2 × 2 design used in fMRI, behavioral and EEG analyses. **(D)** Congruency ratings: the [CO_2_+Orange] mixture was rated as significantly more congruent than [CO_2_+Rose] in “pair rating” and “mixture rating” paradigms. Bars represent s.e.m. ^*^p < 0.05.

The stimuli were rose odor (“Ros,” phenyl ethyl alcohol, 20%, Aldrich Chemie GmbH, Riedstraße 2, Stauheim, Germany; CAS # 60-12-8), orange odor (“Ora,” 20%, Orange aroma oil; Frey and Lau, Henstedt-Ulzburg, Germany), carbon dioxide (“CO2,” 40%, Praxair, Dresden, Germany), an incongruent mixture of rose odor + CO_2_ (“Inc,” 20 + 40%) and a congruent mixture of orange odor + CO_2_ (“Cong,” 20 + 40%) (Figure 1A). Stimuli were mixed before dilution, so that the number of molecules per odorant could be presumed to be identical in the mixtures and in the individual stimuli.

A Burghart OM6b pulsed olfactometer was used to deliver rectangular-shaped chemical stimuli with controlled stimulus onset. Mechanical stimulation was avoided by embedding the stimuli in a constant flow of odorless humidified air at controlled temperature (80% relative humidity; total flow 6 L/min; 36°C) (Kobal, 1981). Prior to the experiment, subjects were trained in the lab to breathe through the mouth without concomitant nasal airflow (velopharyngeal closure; Kobal, 1981) in order to avoid respiratory airflow in the nasal cavity during the chemosensory stimulation. A thermally insulated Teflon™ cannula directed the gaseous stimulus from the olfactometer to the subject's nose in the MRI and EEG rooms.

### fMRI experiment

The study started with the fMRI experiment, which was performed on a 1.5 Tesla MR-scanner (Siemens Sonata, Erlangen, Germany) and lasted approximately 60 min (from the arrival to the departure of the subject). Unlike the EEG experiment, which could be performed in a single session, the fMRI study was divided into 5 functional sessions to allow participants to take a break from the noisy fMRI environment every 5 min. Sessions were randomized, one per stimulus condition: “CO_2_,” “Ros,” “Ora,” “Inc,” and “Con.” Each functional session in turn comprised 6 on/off-blocks, with 24-s blocks presented alternately in the On (stimulus-on) and Off (stimulus-off or “Air”) conditions (Figure 1B). During the “On” conditions (lasting 24 s), odorants were presented 8 times, for 1 s followed by no-odor diffusion for 2 s. The fMRI data were collected in 96 volumes/session with a 36 axial-slice matrix 2D SE/EP sequence (Matrix: 64 × 64; TR: 3 s; TE: 35 ms; FA: 90°; voxel size: 3 × 3 × 3.75 mm). Total duration of the functional sessions was 24 min. In the 6 min immediately following, a high-resolution T1-weighted image of the brain (3D IR/GR sequence: *TR* = 2180ms/*TE* = 3.93 ms) was acquired.

During the functional sessions, subjects were instructed to breathe through the mouth without concomitant nasal airflow (velopharyngeal closure, as described above), were not cued for any stimulus presentation and were not aware of the identity of the stimuli. They were not asked to perform any detection or cognitive tasks during stimulus presentation. For each session, following the 6 on/off-blocks, participants were asked to evaluate the stimulus in terms of intensity (on a scale from “0” = “not perceived” to “10” = “extremely intense”) and of pleasantness (on a scale from “–5” = “extremely unpleasant” to “+5” = “extremely pleasant”). One intensity rating and one pleasantness rating were collected for each session.

Statistical analysis of fMRI data used SPM8 software (Statistical Parametric Mapping; Wellcome Department of Cognitive Neurology, London, UK) implemented in Matlab 7.1 (MathWorks Inc., Natick, MA, USA). Spatial pre-processing comprised registering, realignment with motion parameters included later in the model, co-registration between functional and structural images, normalization in stereotaxic space, and smoothing by means of a 7^*^7^*^7 mm^3^ FWHM Gaussian kernel (Ashburner and Friston, 2003); first-level statistical analysis was then implemented with canonical hemodynamic response functions. For each subject, the following individual contrasts were performed: [“Odors” vs. “Air”], [“CO_2_” vs. “Air”], [“Cong” vs. “Air”], [“Inc” vs. “Air”], [“Cong” vs. its components], and [“Inc” vs. its components], where “Air” corresponds to the stimulus-off period of each condition.

Group analyses used a random-effects model (Penny et al., 2003). In total 4 types of second-level analysis were performed: (1) [“Odors” vs. “Air”] to examine brain areas responding to odors; (2) [“CO_2_” vs. “Air”] to examine brain areas responding to the pure trigeminal stimulus; (3) [“Cong” vs. “Air”] vs. [“Inc” vs. “Air”] (and vice versa) to examine the differential activation of the congruent and the incongruent conditions; and (4) [“Cong” vs. its components] vs. [“Inc” vs. its components] (and vice versa) to examine the differential activation of the congruent condition (minus its components) and the incongruent condition (minus its component).

Activation coordinates were presented in MNI space. We report here results for brain areas in which a congruency effect had previously been demonstrated, as described in the Introduction: the hippocampus, insula, OFC, prefrontal cortex and cingulate gyrus. The primary olfactory cortex (including olfactory areas, amygdala and entorhinal cortex) and somatosensory areas (post-central gyrus) were also included. Activation loci were thus identified within this brain network of interest, delineated by an inclusive mask created with the WFU PickAtlas toolbox (Maldjian et al., 2003).

Areas of significant activation were identified at cluster level for values exceeding a *p*-value of 0.001 (5 voxels, uncorrected). Additionally, small volume corrections (SVC) were implemented, using coordinates from previously published studies, to determine the significance of predicted peaks in anterior cingulate gyrus (15, 33, 27; –10, 48, 4), posterior OFC (36, 18, –12; –16, 32, –4), prefrontal cortex (57, 36, 9), antero-medial OFC (–3, 39, –18; –12, 42, –18), hippocampus (–27, –12, –24) and insula (–32, 22, –8) (Gottfried and Dolan, 2003; Small et al., 2004; Osterbauer et al., 2005).

For behavioral data, because of the nature of the subjective scale (10-point ordinal), the non-parametric Wilcoxon test was applied to intensity and pleasantness ratings. Data were entered into a 2 × 2 design (Figure 1C) and analyzed in two ways. Firstly, congruent and incongruent stimuli were compared directly, for the bimodal condition (mixtures) on the one hand and the unimodal condition (components) on the other. Thus, for intensity and pleasantness ratings, the following comparisons were performed: [“Cong” vs. “Inc”] (bimodal comparison) and [Sum of “Cong” components vs. Sum of “Inc” components] (unimodal comparison).

Secondly, to enable comparison with the fMRI analyses, the following comparison was made for intensity and pleasantness ratings: [“Cong” vs. sum of its components] vs. [“Inc” vs. sum its components], thereby analyzing differential intensity and pleasantness ratings between the congruent condition (minus its components) and the incongruent condition (minus its components).

### EEG experiment

One to 3 days after the fMRI experiment, participants were asked to take part in an EEG experiment lasting approximately 2 h. The 5 stimulus conditions were presented in random order. Each stimulus was presented 15 times with a stimulus duration of 200 ms and an inter-stimulation interval of 40 s. During the experiment, subjects received white noise through headphones to mask the switching clicks of the stimulation device. To stabilize vigilance, subjects performed a tracking task on a computer screen: using a joystick, they had to keep a small square inside a larger one which moved unpredictably (Bensafi et al., 2007a).

EEG was recorded at positions F3, F4, Fz, C3, C4, Cz, P3, P4, and Pz, of the 10/20 system [referenced against linked earlobes (A1 + A2)] using a 16 channel amplifier (Brain Star AC-2000; Schabert instruments, Röttenbach, Germany). Sintered silver-chlorided silver disc electrodes (electrode diameter, 5 mm) were attached to the cleansed skin (“Skin Pure” prepping cream; Nihon Kohden, Tokyo, Japan) using self-adhesive cream (“EC2 Grass Electrode Cream”; Grass, Warwick, RI, USA).

Eye blinks were monitored via the Fp2 lead. Sampling frequency was 250 Hz. Recording time was 2048 ms per recording (bandpass 0.02–30 Hz, with a pre-trigger baseline period of 530ms). Recordings were additionally filtered off-line (low-pass 15 Hz).

CSERPs were averaged after discarding recordings contaminated by motor artifacts or blinks (>50 μV at Fp2), detected by an experienced investigator. The minimum number of trials for each condition remaining after artifact rejection was *n* = 8 (Kobal, 1981; Hummel and Kobal, 2001). Peak amplitudes and latencies (N1 and P2) were measured heuristically by an experienced observer using EPEvaluate 4.2.2 software (Kobal, Erlangen, Germany). To enable comparison with the behavioral and fMRI data, four experimental conditions were included in the 2 × 2 design (Figure 1C): “Cong,” “Inc,” “Components of Cong” (i.e., “CO_2_” and “Ora”) and “Components of Inc” (i.e., “CO_2_” and “Ros”). Because of the continuous nature of the measurements, these conditions were then entered into ANOVAs (rather than non-parametrical tests) including “modality” (2: unimodal, bimodal) and “congruency” (2: incongruent, congruent) as within-subject factors. Additionally a within-subject “electrode” factor (9: Cz, C3, C4, Fz, F3, F4, Pz, P3, P4) was included in the analysis. This 2 × 2 × 9 ANOVA was performed for the amplitudes and latencies of both the N1 and P2 components. The sum of the components was calculated on N1 and P2 amplitudes (to examine a potential hyperadditivity effect), and the mean of the components was calculated for N1 and P2 latencies (as there was no reason to assume any additive effect for EEG latencies).

### Control for perceptual congruency

To ensure that the two mixtures were indeed rated as congruent and incongruent by subjects, a psychophysical study was performed in a separate set of 13 healthy subjects (27.77 ± 3.39 years; 5 men) with normal sense of smell [ascertained by the “Sniffin' Sticks” test (Hummel et al., 1997)]. Here, congruency was assessed on two protocols. Firstly, participants were asked to rate the congruency of two stimuli presented separately (“pair ratings”): CO_2_ was presented first (for 200 ms), followed by a rest period of 10 s, followed by the smell of orange or rose (for 200 ms). Participants had to estimate the congruency between the two stimuli after delivery of the second one (the odor). Instructions were: “You will be presented two stimuli one after the other. Your task will be to estimate how these 2 stimuli match or are congruent: i.e., how far they can be associated in real life (in nature, food, drink, perfume, an object, etc.). To this end, please use the following scale: 0 (no association, match or congruency) to 10 (very associated, matched, congruent).” Each pair of stimuli (CO_2_ followed by orange, or CO_2_ followed by rose) was presented 5 times. The 10 trials were presented in random order with a 1-min interval between pairs of stimuli.

Secondly, subjects were asked to evaluate the congruency between stimuli presented together in mixtures (“mixture ratings”): CO_2_ presented simultaneously with the smell of orange or rose (for 200 ms). The instructions were: “You will be presented a mixture composed of carbon dioxide plus a smell. Your task will be to estimate how far these 2 stimulations match or are congruent and how far they can be associated in real life (in nature, food, drink, perfume, an object, etc.). To this end, please use the following scale: 0 (no association, match or congruency) to 10 (very associated, matched, congruent).” Each mixture (CO_2_+Orange or CO_2_+Rose) was presented 5 times. The 10 trials were presented in random order with a 1-min inter-stimulus interval. All stimuli and mixtures were presented at the same concentrations as in the main study.

As expected, results revealed that the mixture composed of CO_2_+Orange was rated as significantly more congruent than the mixture composed of CO_2_+Rose in both the first paradigm (pair ratings; *p* < 0.01, Wilcoxon test) and the second paradigm (mixture ratings; *p* < 0.03, Wilcoxon test) (Figure 1D).

## Results

### fMRI experiment

Confirmatory analyses examined the main effect of odors and trigeminal stimuli. For odors, the odorant conditions were summed and contrasted with their odorless baseline conditions. Activity was observed in the piriform cortex and inferior frontal gyrus (Supplementary table 1; Supplementary figure 1). The same analysis was performed for the pure trigeminal stimulus (carbon dioxide), and results revealed neural activity in the inferior, middle and superior frontal gyrus, pre- and post-central gyri, cingulate gyrus, frontomarginal gyrus and insula (Supplementary table 1; Supplementary figure 1). These findings replicate previous studies [see Albrecht et al. (2010); Seubert et al. (2012) for reviews] and indicate that our methodology did induce neural activation in the olfactory and trigeminal systems.

**Figure 2:**
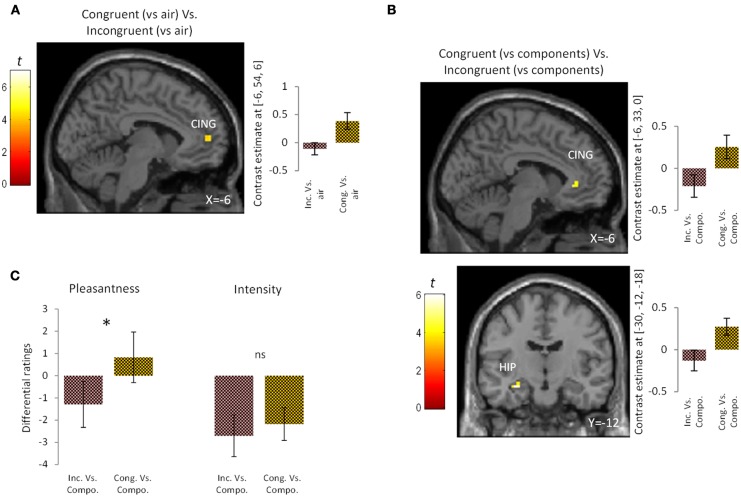
**Brain activation and perceptual ratings. (A)** Brain activation and contrast estimates in response to the congruent mixture (vs. air) vs. the incongruent mixture (vs. air): responses were observed in anterior cingulate cortex (CING, *p* < 0.05 SVC). **(B)** Brain activation and contrast estimates in response to the congruent mixture (vs. its components) vs. the incongruent mixture (vs. its components): responses were observed in the hippocampus (HIP, *p* < 0.05 SVC) and anterior cingulate cortex (CING, *p* < 0.05 SVC). **(C)** Differential ratings, showing pleasantness and intensity ratings for the congruent and incongruent mixtures vs. their individual components (Compo). ^*^p < 0.05; ns = non-significant difference at the 0.05 threshold. Bars represent s.e.m.

To examine the effect of congruency in the perception of bimodal olfacto-trigeminal mixtures, two types of analysis were performed. First, the activation induced by the congruent mixture ([“Cong” vs. “Air”]) was compared to that resulting from the incongruent mixture ([“Inc” vs. “Air”]). Results revealed significant activation in the anterior cingulate gyrus (*p* < 0.05 after SVC) (Figure 2A; Table 1). The opposite contrast (incongruent vs. congruent), on the other hand, did not show any significant activation. In the second analysis, the activation induced by the congruent mixture minus its components was compared to that resulting from the incongruent mixture minus its components. In this case, significant activation was observed in the hippocampus (*p* < 0.05 after SVC), accompanied by additional activation in the anterior cingulate gyrus bordering the upper part of the posterior orbitofrontal gyrus (*p* < 0.05 after SVC) (Figure 2B; Table 1). The opposite contrast, on the other hand, did not show any significant activation.

**Table 1:** **Activation in response to (1) the congruent mixture (vs. air) vs. the incongruent mixture (vs. air) and (2) to the congruent mixture (vs. its components) vs. the incongruent mixture (vs. its components)**.

	***K***	***T-*value**	***x***	***y***	***z***	**Brain areas**
Congruent mixture (vs. air) vs. the Incongruent mixture (vs. air)	7	4.31	−6	54	6	Cingulate gyrus
Congruent mixture (vs. components) vs. the Incongruent mixture (vs. components)	7	6.02	−30	−12	−18	Hippocampus
	5	4.55	−6	33	0	Cingulate gyrus

K is the cluster size. Statistical t-values are presented. MNI coordinates of activated brain areas are presented in x, y, and z.

At a perceptual level, the congruent bimodal mixture was perceived as more intense than the incongruent bimodal mixture (mean ± s.e.m.: congruent mixture = 7.47 ± 0.59; incongruent mixture = 6.29 ± 0.59; *p* = 0.05) and as more pleasant (mean ± s.e.m.: congruent mixture = 1.64 ± 0.58; incongruent mixture = −0.23 ± 0.65; *p* < 0.03). Comparison of the unimodal components of the congruent and incongruent stimuli revealed a trend toward greater intensity for the congruent vs. the incongruent components (mean ± s.e.m.: congruent components = 9.64 ± 0.73; incongruent components = 9.00 ± 0.80; *p* = 0.06) but no significant difference in pleasantness (mean ± s.e.m.: congruent components = 0.82 ± 1.10; incongruent components = 1.06 ± 0.84; *p* = 0.83). Finally, direct comparison between the congruent condition (minus its components) and the incongruent condition (minus its components) revealed that the congruent mixture was perceived as more pleasant (*p* < 0.05) but not more intense (*p* = 0.53) than the incongruent mixture (Figure 2C).

### EEG experiment

**Figure 3:**
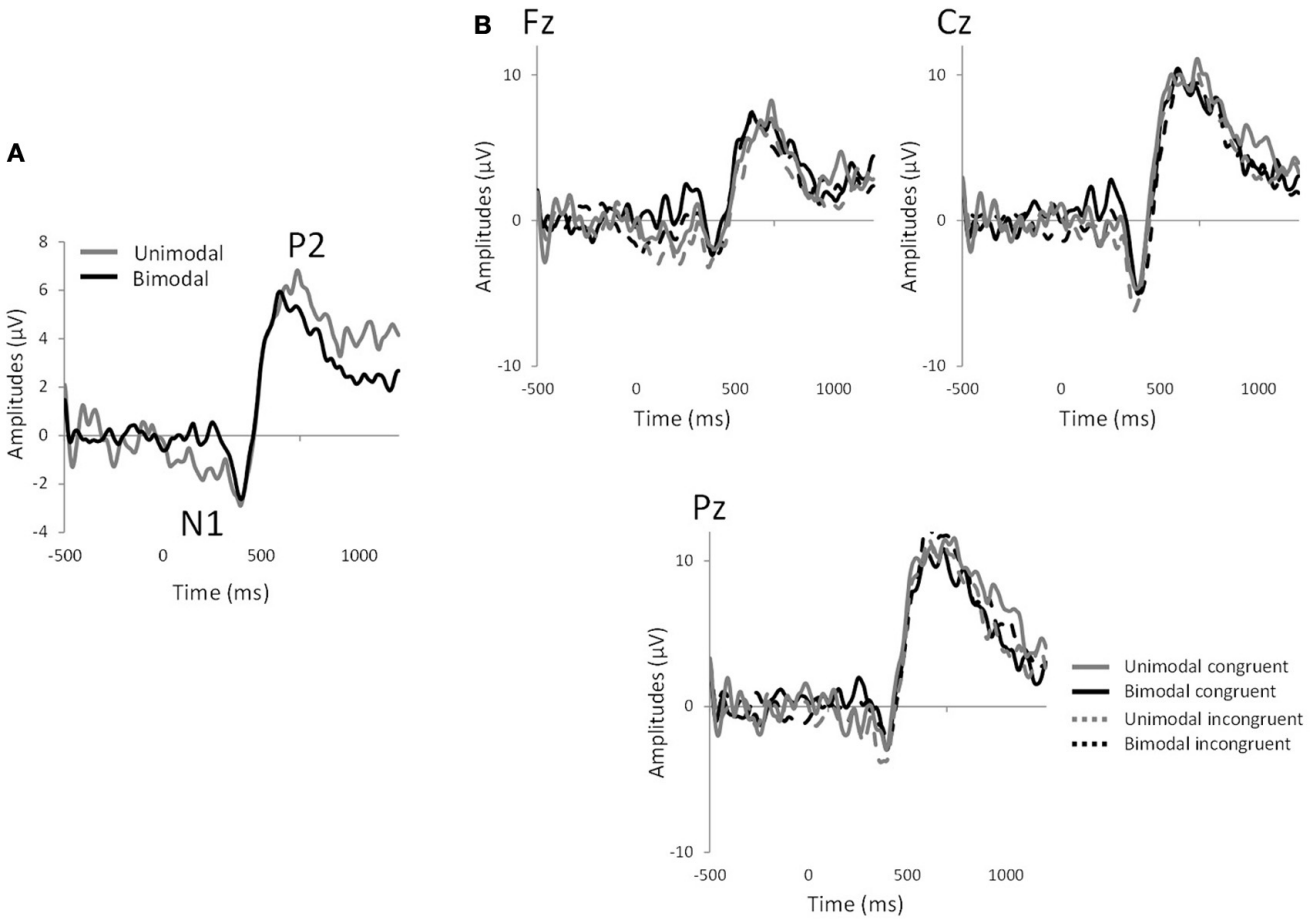
**EEG measurements. (A)** Grand average of chemosensory event-related potentials averaged across all electrodes and subjects for the unimodal and bimodal conditions, showing the N1 and P2 components. **(B)** Chemosensory event-related potentials for the 4 experimental conditions: bimodal congruent mixture (CO_2_Orange), bimodal incongruent mixture (CO_2_Rose), unimodal congruent components (CO_2_+Orange) and unimodal congruent components (CO_2_+Rose) in Fz, Cz, and Pz electrode sites.

Significant effects of “electrode” were observed for N1 latency [*F*_(8, 128)_ = 2.59, *p* < 0.01] and P2 amplitude [*F*_(8, 128)_ = 2.63, p < 0.02]. In all the analyses, no significant effect of congruency was observed (*p* > 0.05 in all cases). However, significant effects of “modality” were observed for N1 amplitude [mean ± s.e.m.: unimodal = −10.38 ± 1.23; bimodal = −5.63 ± 0.89; *F*_(1, 16)_ = 28.11, *p* < 0.0001], N1 latency [mean ± s.e.m.: unimodal = 439.69 ± 13.77; bimodal = 382.49 ± 11.00; *F*_(1, 16)_ = 17.48, *p* < 0.0008] and P2 latency [mean ± s.e.m.: unimodal = 564.56 ± 18.05; bimodal = 495.46 ± 15.01; *F*_(1, 16)_ = 23.38, *p* < 0.0003], reflecting the fact that the bimodal mixtures evoked shorter N1 and P2 latencies and smaller N1 amplitudes than their individual components (Figure 3). Interactions between factors did not reach significance (*p* > 0.05) except for P2 latency, where an “electrode”-by-“congruency” interaction was observed [*F*_(8, 128)_ = 2.12, *p* < 0.04]. Nevertheless, paired comparison within each electrode site did not reveal any effect of congruency (*p* > 0.05 in all 9 cases).

## Discussion

The aim of the present study was to explore the effect of congruency on the perception and neural responses induced by combined odor-trigeminal stimuli. The first result of interest was that congruency affected the perceptual ratings of bimodal chemosensory stimuli: the congruent mixture was perceived as more pleasant and more intense than the incongruent mixture. Analysis of the cumulative effect of the congruent and incongruent mixtures compared to their individual components showed that the two mixtures differed in terms of pleasantness but not intensity. These findings agree with a large set of psychophysical experiments showing that congruency enhances the intensity and/or pleasantness of bimodal stimuli (Schifferstein and Verlegh, 1996; De Araujo et al., 2003; Small et al., 2004; Seo et al., 2010; Seo and Hummel, 2011). Analysis of chemosensory event-related potentials revealed that both binary mixtures (congruent and incongruent) induced shorter N1 and P2 latencies and smaller N1 amplitudes compared to their individual components. Although studies in the non-chemosensory domain showed an effect of congruency on the N400 component of event-related potential (Kutas and Federmeier, 2011), the present EEG study did not show any direct temporal difference between congruent and incongruent mixtures. Because CSERPs were recorded from a small number of electrodes, it is not unlikely that spatial differences exist but are not reflected by our EEG measures. Indeed, fMRI data revealed that congruency affected the spatial processing of chemosensory cross-modal integration.

A major result of the present study was the differential activation pattern seen during perception of the congruent compared to the incongruent mixture, notably in the cingulate gyrus and hippocampus. Past and more recent studies have revealed neural activity in cingulate gyrus in response to olfactory and trigeminal stimuli (Iannilli et al., 2007; Bensafi et al., 2008; Seubert et al., 2012), and a previous study suggested that the cingulate cortex is a multi-integrative structure in processing chemosensory stimuli (Small et al., 2004). Anatomically, cyto-architectural studies of the cingulate gyrus support a multiple-region model, with anterior, middle and posterior parts (Vogt, 2005). The functioning of these sub-regions is not homogeneous and their involvement in cross-modal integration may differ according to modality. Klasen and colleagues showed that the ventral posterior cingulate cortex was activated during integration of congruent audiovisual stimuli (Klasen et al., 2011), while others showed that pleasant olfacto-verbal associations activated the anterior part of the cingulate cortex (De Araujo et al., 2005). The present findings are in line with the above results, highlighting a role of this brain area in binding olfactory and trigeminal representations of environmental objects.

With regard to the hippocampus, many investigations revealed a functional role of this brain area in chemosensory processing, especially odor processing. For example, positive correlation was observed between the volume of the hippocampus and odor threshold performances of healthy subjects (Smitka et al., 2012). Moreover, compared to sighted volunteers, congenitally blind subjects showed stronger hippocampal activation during a detection task (Kupers et al., 2011). The hippocampus is also involved in more cognitive olfactory tasks. For instance, hippocampal activity increases significantly as a function of odor identifiability (Kjelvik et al., 2012) and amnesic patients with atrophy of the hippocampus are impaired for odor-place associative memory (Goodrich-Hunsaker et al., 2009). The role of the hippocampus in binding between different stimuli has been established by previous studies (see for reviews Squire et al., 2004; Eichenbaum et al., 2007). For example, in the non-chemosensory domains, it has been observed that memory for congruous events, defined as events whose constituent elements match along particular attributes, recruit a network involving the inferior frontal gyrus and the hippocampus (Staresina et al., 2009). Moreover, the hippocampus was shown by Gottfried and Dolan to be activated by congruent pairs of visual and olfactory stimuli, suggesting that it is a key component of the network underlying the binding of semantic information from different modalities (Gottfried and Dolan, 2003). Combined with the above, our results therefore suggest that emotional information during cross-modal integration of congruent pairs of odors and trigeminal stimuli also merges in the hippocampus, this area being potentially involved in binding the associations of both unimodal stimuli to form a harmonious mnemonic representation.

As mentioned in the introduction, such harmony or congruency is linked to familiarity, explaining why a bimodal stimulus comprising a painful trigeminal stimulus and a pleasant odor is appetitive for the tested individuals: even when one unimodal stimulus (here, intranasal carbon dioxide) arouses a sensation of pain, this intrinsically painful feature becomes part of the integrated percept of a familiar object. To sum up, congruency is likely the fruit of experience and culture, since it is based on the formation of previous associations in particular contexts; congruency effects influence the perceptual, emotional and cognitive processing of sensory stimuli, probably via expectancy (see Schifferstein and Verlegh, 1996). It is known that, when expectancies are evoked by colors (Osterbauer et al., 2005), tastants (Yeomans, 2006; Barkat et al., 2008), verbal labels (Herz, 2003; Bensafi et al., 2007b, 2013; Rinck et al., 2011) or sounds (Seo and Hummel, 2011), they can alter the intensity and/or pleasantness ratings of chemosensory stimuli. The present study is the first to highlight such effects of congruency on odor-trigeminal integration.

Although the present study provides evidence for neural modulation by congruency, some of the findings deserve discussion. Certain aspects might be explained not only by the integration of the two harmonious congruent stimuli but also by probability summation mechanisms. Evidence that perceptual response is higher in a bimodal condition compared to unimodal conditions does not necessarily mean that the two stimuli interact perceptually: instead, it may be that two sensory stimuli are more likely than single signals to induce perceptual response. This concept was originally introduced for sensory thresholds, and recent studies suggest that it should be considered for detection of bimodal olfacto-gustatory stimuli (Veldhuizen et al., 2011). In the present case, nevertheless, the stimulations were relatively intense and no additive effect on perceived intensity was observed. On the contrary, the two mixtures were significantly less intense than the sum of their components, reflecting a hypo-additivity effect. Moreover, at a neural level, activation always resulted from a comparison between two bimodal stimuli.

In conclusion, the present study showed that a congruent association between an odor and a trigeminal stimulus was perceived as more pleasant than an incongruent association. This behavioral effect was associated with enhanced neural activity in the hippocampus and anterior cingulate gyrus, indicating that these brain areas mediate reactivation of pleasant and congruent olfactory-trigeminal associations. Taken together, these results are in line with the general view that, when a stimulus is encoded, the percept that emerges does not simply come from hierarchical processing in a single modality, from sensory transduction to the creation of a single mental representation: rather, chemosensory integration depends on other available stimuli (olfactory and trigeminal in the present case), and congruency between these cues is a prominent factor in the emotional perception of objects, as observed for the integration of smells with other sensory stimuli.

### Conflict of interest statement

The authors declare that the research was conducted in the absence of any commercial or financial relationships that could be construed as a potential conflict of interest.
