# Supplementary material for: Cross-modal integration of emotions in the chemical senses
Source: Front Hum Neurosci. 2013 Dec 20;7:883. doi: 10.3389/fnhum.2013.00883 (PMC3868915; doi:10.3389/fnhum.2013.00883)
Supplement: Supplementary file 1 [file Presentation1.PDF]

Supplementary information

Bensafi et al. Cross-modal integration of emotions in the chemical senses

Supplementary Figure 1. Brain activation in response to olfactory and trigeminal stimuli. Activation in response to odors was found in the piriform cortex and inferior frontal gyrus (a). Responses to the pure trigeminal stimulus (carbon dioxide) were found in the insula, cingulate gyrus, inferior, middle and superior frontal gyrus, pre- and post-central gyri and frontomarginal gyrus (b).

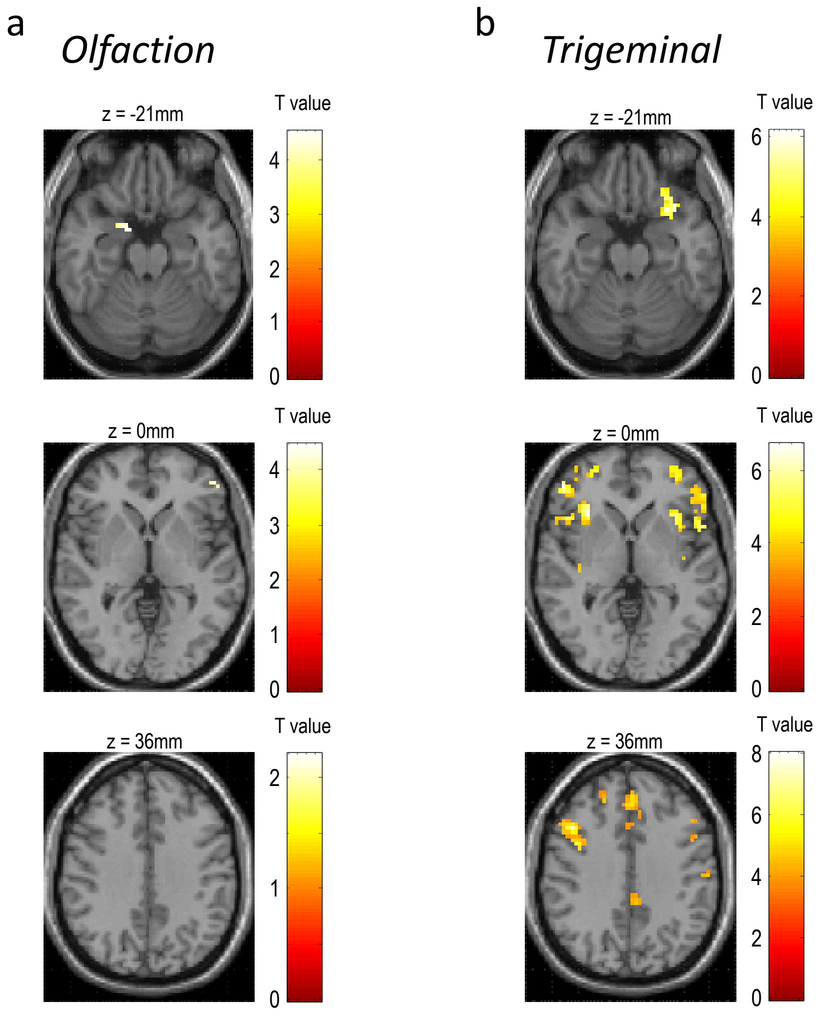

Supplementary Table 1. Activation in response to Olfactory (vs. air) and Trigeminal (vs. air) stimuli. K is the cluster size. Statistical t values are presented. MNI coordinates of activated brain areas are presented in x, y, and z.

	K	t value	x	y	z	Brain areas
Olfactory (vs. air)	9	8.20	15	0	-21	piriform cortex
	5	8.14	-48	45	-3	inferior frontal gyrus
Trigeminal (vs. Air)	841	8.01	51	39	3	inferior frontal gyrus
		6.86	45	21	42	medial frontal gyrus
		6.78	51	12	30	inferior frontal gyrus
	697	6.76	-57	0	15	pre/post central gyrus
		5.81	-54	15	21	inferior frontal gyrus
		5.14	-42	36	27	medial frontal gyrus
	26	5.45	3	3	30	cingulate gyrus
		4.67	3	-6	30	cingulate gyrus
	62	3.95	0	39	39	superior frontal gyrus
		5.21	3	24	42	superior frontal gyrus
		5.20	-6	30	36	superior frontal gyrus
	6	4.91	-12	57	-9	frontomarginal gyrus
	38	4.05	-36	-6	6	insula
		5.15	-39	-21	12	insula
		4.88	-45	-12	3	insula
	16	4.78	-3	-36	33	cingulate gyrus
	19	4.62	-33	54	0	frontomarginal gyrus
	8	4.61	60	0	18	pre-central gyrus
	17	4.58	-12	39	24	cingulate gyrus and superior frontal gyrus
	6	4.50	36	-18	3	insula
	6	4.45	42	54	-3	inferior frontal gyrus
	5	4.31	36	-18	12	insula
	5	4.14	-57	-18	36	post-central gyrus
	7	4.01	-21	51	24	supra frontal gyrus
	5	3.98	6	-27	30	cingulate gyrus
	5	8.20	33	3	9	insula
	6	8.14	3	39	15	cingulate gyrus
